# Supplementary material for: Health systems’ capacity in availability of human resource for health towards implementation of Universal Health Coverage in Kenya
Source: PLoS One. 2024 Jan 30;19(1):e0297438. doi: 10.1371/journal.pone.0297438 (PMC10826929; doi:10.1371/journal.pone.0297438)
Supplement: Attachment — Submitted filename: Reviewers Comments_HRH in Kenya_PONE-D-23-11190R1.pdf [file pone.0297438.s002.pdf]

Reviewer's comments_PONE-D-23-11190R1

<u>Health systems' capacity in availability of human resource for health towards implementation of Universal Health Coverage in Kenya</u>

<u>General Comments</u>

In the abstract and introduction section, Kenya's challenges with the availability of HRH is presented but it is not clear how this affects the attainment of Universal Health Coverage (UHC).

In lines 71-73 on page 3, the following sentence is hanging: "In 2017, building on the Road map for scaling up HRH for improved health service delivery in the African Region 2012–2025 that was adopted by the Sixty-second session of the Regional Committee."

In lines 119-123 on page 5. The interviewees are listed as the study population! I think this is not correct. I thought the study population should be the total number of health facilities in the study areas.

In lines 137-138 on page 6, it is stated that "Informants were purposively selected from various categories. A total of 36 FGDs and 30 IDIs per county were targeted." What informed the targeting? How many people were in each FGD? Could you describe the FGD process in terms of recruitment, discussion process, number of iterations, etc.

In lines 140-143 on page 6. The differences between the outcomes and exposures are not clear. The sentences defining these terms seem to be the same to me.

In lines 144-145 on page 6, the definition for the predictors is provided. However, these are not used to interpret the results and in the discussion. The authors could use the predictors to explain the factors that influence the availability and distribution of HRH and the implementation of UHC in Kenya.

On page 8 (lines 188-208), the section on ethical consideration is very long. This could be cut to three short sentences.

In the results section, the information provided from lines 212-234 (page 9) could be cut and taken to the methodology section.

Please add p-values in the tables where necessary.

Table 4 could be summarised. Too many details are provided most of which could be summarised in a short table. The full table could be part of the supplementary documents.

Results from the quantitative and qualitative components of the study are presented separately. They could be presented together and/or alongside to improve interpretation.

Overall, the discussion is very long and there is inadequate triangulation of the results from the study with other studies. The authors must ensure that the results are linked to the objectives of the study, sufficiently interpreted and triangulated. In most cases, the previous studies and examples that have been used in the discussion section to interpret the results are not linked to actual findings from the study.

The conclusion as at the end of the article is very too long and not sufficiently linked to the objectives of the study. The authors seem to be presenting the results again rather than concluding. The authors

need to prepare a conclusion with only 2-3 sentences linked to the objectives of the study. The recommendations and areas for future research also need to be provided and linked to the conclusion of the study.

Limitations

About three limitations have been provided but the authors have not explained how these limitations were addressed or mitigated in the study. The authors must address this issue. Secondly, the study didn't look at the effect or impact of non-availability of HRH in the achievement on UHC. On the other hand, increased availability of health workers (including optimal distribution and skills-mix) is a necessary but not the absolute condition for success or progression to UHC. What if the conditions of service are poor and the salaries are low? The health workers could be unproductive? What if the health workers have low skills? What if the other health systems inputs like drugs, vaccines, medical equipment, etc are not available?

The analysis also misses a budget and futuristic financing landscape analysis to determine if meeting the staffing norms is feasible in view of the financial resources that the Kenyan government has now and in future. Lastly, the study also misses an analysis of the HRH training outputs and health workers currently not employed to fill the set staffing norms. This analysis could complement the analysis on the staffing gaps vis-à-vis the availability of labour to fill the gaps, and government's financing ability/capacity to employ.
