# Supplementary material for: Health systems’ capacity in availability of human resource for health towards implementation of Universal Health Coverage in Kenya
Source: PLoS One. 2024 Jan 30;19(1):e0297438. doi: 10.1371/journal.pone.0297438 (PMC10826929; doi:10.1371/journal.pone.0297438)
Supplement: Attachment — Submitted filename: Response to Reviewers.docx [file pone.0297438.s001.docx]

**Response to Reviewers**

| **Editor’s Comment** | |  |
| --- | --- | --- |
| **Comments** | **Response** | **Page** |
| Please ensure that your manuscript meets PLOS ONE's style requirements | Edited to meet PLOS ONE's style requirements | Whole |
| We note that the grant information you provided in the ‘Funding Information’ and ‘Financial Disclosure’ sections do not match. | Edited to match | 24 |
| Update your Data Availability statement to reflect the information you provide. | Ready to provide any data related to the manuscript | Cover letter |
| You may seek permission from the original copyright holder of Figure 1 to publish the content specifically under the CC BY 4.0 license. | Removed the Map | 5 |
| **Reviewer #1** | |  |
| **Comments** | **Response** | **Page** |
| The study design and methodology should have been more detailed and transparent, including the sampling strategy and the criteria used to select the study sites. | Revised | 4-5 |
| The article lacks a clear description of the statistical methods used for data analysis and does not provide any information on the reliability and validity of the data collection tools. | Revised | 7-8 |
| The article fails to provide a comprehensive analysis of the factors influencing the availability of HRH in Kenya. The article highlights the shortage and maldistribution of HRH in the country, but it does not explore the underlying causes of these challenges. | Stated as study limitation | 24 |
| The article lacks a clear discussion on the potential implications of the findings for the implementation of UHC in Kenya. | Revised | 20-22 |
| The article could have provided a more critical reflection on the limitations and implications of the study. | Revised | 20-22 |
| **Reviewer #2** | |  |
| **Comments** | **Response** | **Page** |
| The general format of paper needs to revise and adjust based on essentials of Journal. | Revised | Whole |
| Abstract: The abstract needs a purposeful revising in terms of both; form and content | Revised | 1-2 |
| Add appropriate key words based on MeSH categories of PubMed | Revised | 2 |
| Introduction: Introduction need to revision that mainly focus on the limitations/ gaps of evidence and justification of present study. | Revised | 4 |
| Methods: Refer to main characteristics of original survey and cite to its protocol study and related extracted papers. | Revised | 4 |
| Results: The visualization of results needs to more attempts for better attraction | Figure 1 and 2 | 11 |
| Results: Try to present analytical results and not limit the report to the description of indicators | Revised | 10-19 |
| Discussion: Complete the discussion by comparing the findings with those of other studies and applying an analysis of the causes of the differences in results |  |  |
| Discussion: Point to many solutions to delimit the challenges ahead as practical implication of findings. | Revised | 20-21 |
| Conclusion should be based on the net findings and proposing some general suggestions would not be practical. | Revised | 22-23 |
