# Supplementary material for: Health systems’ capacity in availability of human resource for health towards implementation of Universal Health Coverage in Kenya
Source: PLoS One. 2024 Jan 30;19(1):e0297438. doi: 10.1371/journal.pone.0297438 (PMC10826929; doi:10.1371/journal.pone.0297438)
Supplement: Attachment — Submitted filename: Comments_PONE.docx [file pone.0297438.s003.docx]

**Comments**

The manuscript addresses an important topic in health service delivery and universal health coverage reforms, currently underway in Kenya. The authors can make the paper better by institution the following revisions:

Title: In the affiliations, Kenya Medical Research Institute and KEMRI are used, but not linked. A person not familiar with the setting may conclude they are two different institutions.

Abstract: the abstract should always be able to stand on its own, that’s what majority of people will read anyway.

HRH is used but not represented in full. It’s always good practice to put abbreviations in full at first use.

Sampling: if the facilities were randomly selected, what about the counties? For the UHC counties it is understandable since the authors did a census, but what about the other eight?

Data collection (quantitative) approach is not clear. Whas it interviewing, observational checklists, document search (staff records). Who was interviewed? This is important since you don’t expect every person to know all the details of healthcare workers in a facility.

The authors state that the general service availability index was measured using five tracer items. What are these trace items? They need to be named.

UHC: see comment on abbreviations above.

Qualitative data collection: what was the study population, for FGDs and for key informant interviews?

Results: check you capitalizations. Capitalization of ‘healthcare’ in line 33 is unnecessary.

The statement in lines 33 and 34 stating no difference between the two groups of counties need to be supported with the relevant statistics, for the reader to interpret for themselves.

Line 38 talks of ‘health worker skill set’: what does this mean? Is it a defined variable in the methods?

KEPH: see comment above on abbreviations. Also briefly define what KEPH levels are about, for a general reader.

There are no results in the abstract from the qualitative component of the study. Wasn’t it introduced as a mixed methods study, with FGDs and KIIs?

**Introduction:**

Line 95 and 96: Instead of putting objectives as a sub-heading, the authors can frame it as the last sentence in the introduction.

Methods:

Is it methodology or methods? Isn’t methodology the study of methods, on a light note?

Study sites: the selection of ‘near’ and ‘far’ needs more explanation. Does near mean bordering? What about far? How far? In a different region? I understand Kenya has around 10 geopolitical regions, and then the 47 counties.

In addition, was the near and far selection random, purposive? I mean a typical county could have four or five neighboring counties. So, how was one selected? These details are quite critical, so the authors need to describe them in detail to aid in interpreting their findings.

How were the 791 facilities distributed in the 12 counties?

**Results**

Is there any reason for naming percent throughout the results, instead of using the % symbol?

Lines 220 through 231 is supposed to be in the methods.

Table 1: what do the highlighted rows represent? Again, tables should be able to stand-alone. If additional details are necessary, they are added as a footnote to enable the reader to interpret the table.

Line 243: there’s a full-stop at the wrong place. Also, simplify the comparison between public and non-public facilities, it seems confusing.

The statement in line 255 needs to be in the discussion.

Line 257; check capitalizations. Also, if possible, avoid ‘respectively’ in your comparisons.

Line 263: avoid phrases like ‘crystal clear’; they have no relevance in scientific writing.

Lines 280-282; discussion. Same case applies to 290-294.

I think table 4 should be either summarized or be a supplementary.

Qualitative: although the aim is to capture the verbatim responses from participants, the authors can assist in re-phrasing it for readers to understand. For instance, corona pandemic, COVID-19 can be added in parentheses. This should be done in a way that it doesn’t distort the original message.

**Discussion**

Line 370: the authors have used compare in the same sentence twice.

Line 389: Be consistent with your spacing.

Lines 398 and 399: It is advisable to put reference at the end of the sentence, not in the middle. This makes reading easier.

Line 404: Were primary care facilities defined in the methods, and then linked with the KEPH structure/ If not, please review.

**Conclusion**

The conclusion is too long and feels like a repetition of the discussion. The authors should use this section to summarize their key findings and through in some recommendations.

Study limitations: this should come before the acknowledgements.
