# Supplementary material for: Health systems’ capacity in availability of human resource for health towards implementation of Universal Health Coverage in Kenya
Source: PLoS One. 2024 Jan 30;19(1):e0297438. doi: 10.1371/journal.pone.0297438 (PMC10826929; doi:10.1371/journal.pone.0297438)
Supplement: Attachment — Submitted filename: Response to Reviewers.docx [file pone.0297438.s004.docx]

**Response to Reviewers**

| **Reviewer #3:** | |  |
| --- | --- | --- |
| **Comments** | **Response** | **Page/Line** |
| Define all concepts, preferably quoting government documents, and place them in context. Expand all abbreviations - Use the expanded version at the first instance, immediately followed by the abbreviation in brackets; and then start using the abbreviation thereafter. This included even UHC! Do not leave readers to guess! Expand abbreviations KEPH, HRM! | Revised | Pg.1  line 16  line 19  line 28 |
| Methodology Define concepts - KEPH Levels 2, 3, 4, etc. What do they mean, how do they differ from each other? Quote from Ministry of Health (MoH) documents. | Revised | Pg. 4-5  line 108-112 |
| Define UHC pilot counties and non-UHC pilot countries; quote from MoH document. Explain the extent of funding provided to facilities. | Revised | Pg. 5  line 115-125 |
| What is meant by 'targeted'? e.g., Page 5, Line 131; and elsewhere. | Means sampled | Pg. 6  line 138 |
| Page 6; Line 791 (sampling) - How did the authors arrive at this number? Procedure and criteria? | Revised | Pg. 6  line 143-144 |
| Why simple random sampling, and not stratified random sampling - if KEPH level distribution of the facilities are available? How was proportional representation ensured? | To give them equal chance of selection. | Pg. 6  line 133-138 |
| Lines 223-4: If this tool is a published document, give reference and give bibliographic information under 'References'. | Tool reference inserted | Pg. 9  line 231 |
| In what language was the questionnaire (and informed consent) administered to various categories of respondents? Was the questionnaire translated into local language (and back-translated)? | Back-translated into local language | Pg. 8  line 212-213 |
| Page 5, Line 119 - Have a closer look at this heading: study population. Where are you describing your 'respondents', how many from each category; and the method by which you arrived at these numbers? | Survey tool was administered to facility in-charge and other population are for qualitative arm | Pg. 5  line 127-130 |
| What is meant by county in Kenyan parlance? Equivalent to a district, OR State / Province? | Equivalent to a district | No Pg. |
| What is the total number of counties? How many were selected? How? Criteria? Consider drawing a single diagram showing the entire sampling procedure so that the whole process could be captured from a single canvass. | Revised | Pg. 5  line 115-125 |
| Have a closer look at lines 45-47 on page 2 (anything missing?). | Revised | Pg. 2  line 52-53 |
| Page 4; Lines 84-84: What is the percentage deficiency? | Revised | Pg. 4  line 91 |
| Results & Discussion: To improve readability and comprehension, in tables, consider writing the norms for each category of staff and against that write the actual number of staff in place, followed by number of vacant posts and percentage deficiency. | Percentage deficiency is the percentage required to achieve 100% on the indicated available percentage | No Pg. |
| What are the reasons for health worker shortages? What are the underlying factors? | No systems of managing resources | Pg. 15  line 326 |
| Student intake for these professional courses that produce these personnel? Government / private break-up? Share of out-migration, and in-migration, especially that of nurses; pay in neighbouring countries vis-a-vis migration? | This was not captured in the research | No Pg. |
| What were the major themes that emerged from content analysis? | Shortage of HRH, need for investment, system to manage limited resources | Pg. 14-15  line 299, 309 and 326 |
| References: Try to include more references to support rationale and findings. What was the search strategy?  If organization is the author, write the name completely in the same order it is written in the source document; do not reformat it into first name, followed by middle and last names (e.g., reference numbers 4 and 14); do not abbreviate the name. (First name, followed by initials is recommended for individual authors, not for organizations). Check names of authors and initials under the list of references. | Revised | Pg. 22-23 |
| General - formatting, etc.  Rephrase some of the sentences.  e.g, Page 11; Lines 260-72: Either write percent in ...... (name of facility), OR write name of facility followed by percent within brackets.  Check grammar: e.g., 'data was'. Spell check the document - using MS Word AND proof-read manually, e.g., Page 19; Line 382: 'per-urban' | Revised | Whole document |
| **Reviewer #4** | |  |
| **Comments** | **Response** | **Page** |
| In the abstract and introduction section, Kenya’s challenges with the availability of HRH is presented but it is not clear how this affects the attainment of Universal Health Coverage, | Revised | Pg. 2-3 |
| In lines 71-73 on page 3, the following sentence is hanging: “In 2017, building on the Road map for scaling up HRH for improved health service delivery in the African Region 2012–2025 that was adopted by the Sixty-second session of the Regional Committee.” | Revised | Pg. 3  line 79-81 |
| In lines 119-123 on page 5. The interviewees are listed as the study population! I think this is not correct. I thought the study population should be the total number of health facilities in the study areas. | Questionnaire was administered to facility in-charges. | Pg. 5  line 127-130 |
| In lines 137-138 on page 6, it is stated that “Informants were purposively selected from various categories. A total of 36 FGDs and 30 IDIs per county were targeted.” What informed the targeting? How many people were in each FGD? Could you describe the FGD process in terms of recruitment, discussion process, number of iterations, etc. | FGD comprises of 8-10 participants. | Pg. 6  line 148-150 |
| In lines 140-143 on page 6. The differences between the outcomes and exposures are not clear. The sentences defining these terms seem to be the same to me. | Revised | Pg. 6  line 152-155 |
| In lines 144-145 on page 6, the definition for the predictors is provided. However, these are not used to interpret the results and in the discussion. The authors could use the predictors to explain the factors that influence the availability and distribution of HRH and the implementation of UHC in Kenya. | Revised | Pg. 6  line 156 |
| On page 8 (lines 188-208), the section on ethical consideration is very long. This could be cut to three short sentences. | Revised | Pg. 8-9  line 206-217 |
| In the results section, the information provided from lines 212-234 (page 9) could be cut and taken to the methodology section. | Revised | Pg. 9-10  line 218-245 |
| Please add p-values in the tables where necessary. | Added | Pg. 10  line 259 |
| Table 4 could be summarized. Too many details are provided most of which could be summarized in a short table. The full table could be part of the supplementary documents. | Revised | Pg. 13-14  line 295-296 |
| Results from the quantitative and qualitative components of the study are presented separately. They could be presented together and/or alongside to improve interpretation. | It was separated in first manuscript but was told to separate by a reviewer | No pg. |
| Overall, the discussion is very long and there is inadequate triangulation of the results from the study with other studies. The authors must ensure that the results are linked to the objectives of the study, sufficiently interpreted and triangulated. In most cases, the previous studies and examples that have been used in the discussion section to interpret the results are not linked to actual findings from the study. | Revised | Pg. 16-18 |
| The conclusion as at the end of the article is very too long and not sufficiently linked to the objectives of the study. The authors seem to be presenting the results again rather than concluding. The authors need to prepare a conclusion with only 2-3 sentences linked to the objectives of the study. The recommendations and areas for future research also need to be provided and linked to the conclusion of the study. | Revised | Pg. 19-20  line 432-449 |
| Limitations  About three limitations have been provided but the authors have not explained how these limitations were addressed or mitigated in the study. The authors must address this issue. Secondly, the study didn’t look at the effect or impact of non-availability of HRH in the achievement on UHC. On the other hand, increased availability of health workers (including optimal distribution and skills-mix) is a necessary but not the absolute condition for success or progression to UHC. What if the conditions of service are poor and the salaries are low? The health workers could be unproductive? What if the health workers have low skills? What if the other health systems inputs like drugs, vaccines, medical equipment, etc are not available?  The analysis also misses a budget and futuristic financing landscape analysis to determine if meeting the staffing norms is feasible in view of the financial resources that the Kenyan government has now and in future. Lastly, the study also misses an analysis of the HRH training outputs and health workers currently not employed to fill the set staffing norms. This analysis could complement the analysis on the staffing gaps vis-à-vis the availability of labor to fill the gaps, and government’s financing ability/capacity to employ. | Revised | Pg. 20  line 455-458 |
|  |  |  |
| **Reviewer #5** | |  |
| **Comments** | **Response** | **Page** |
| Title: In the affiliations, Kenya Medical Research Institute and KEMRI are used, but not linked. A person not familiar with the setting may conclude they are two different institutions.  Abstract: the abstract should always be able to stand on its own, that’s what majority of people will read anyway. | Revised | Pg. 1  line 8 |
| Abstract  HRH is used but not represented in full. It’s always good practice to put abbreviations in full at first use.  UHC: see comment on abbreviations above.  Qualitative data collection: what was the study population, for FGDs and for key informant interviews?  Results: check you capitalizations. Capitalization of ‘healthcare’ in line 33 is unnecessary.  The statement in lines 33 and 34 stating no difference between the two groups of counties need to be supported with the relevant statistics, for the reader to interpret for themselves.  Line 38 talks of ‘health worker skill set’: what does this mean? Is it a defined variable in the methods?  KEPH: see comment above on abbreviations. Also briefly define what KEPH levels are about, for a general reader.  There are no results in the abstract from the qualitative component of the study. Wasn’t it introduced as a mixed methods study, with FGDs and KIIs? | Revised | Pg. 1-2 |
| Introduction:  Line 95 and 96: Instead of putting objectives as a sub-heading, the authors can frame it as the last sentence in the introduction. | Revised | Pg. 4  line 101-103 |
| Methods:  Is it methodology or methods? Isn’t methodology the study of methods, on a light note? | It sound more scientific, on a light note though | No p.g. |
| Study sites: the selection of ‘near’ and ‘far’ needs more explanation. Does near mean bordering? What about far? How far? In a different region? I understand Kenya has around 10 geopolitical regions, and then the 47 counties. In addition, was the near and far selection random, purposive? I mean a typical county could have four or five neighboring counties. So, how was one selected? These details are quite critical, so the authors need to describe them in detail to aid in interpreting their findings. | Revised | Pg. 5  line 115-125 |
| How were the 791 facilities distributed in the 12 counties? | Revised | Pg 6  line 140-141 |
| Sampling: if the facilities were randomly selected, what about the counties? For the UHC counties it is understandable since the authors did a census, but what about the other eight? | Revised | Pg. 5  line 119-125 |
| Data collection (quantitative) approach is not clear. Whas it interviewing, observational checklists, document search (staff records). Who was interviewed? This is important since you don’t expect every person to know all the details of healthcare workers in a facility. | Revised | Pg. 5  line 12-130 |
| The authors state that the general service availability index was measured using five tracer items. What are these trace items? They need to be named | Revised | Pg. 9  line 238 |
| Results  Is there any reason for naming percent throughout the results, instead of using the % symbol?  Lines 220 through 231 is supposed to be in the methods.  Table 1: what do the highlighted rows represent? Again, tables should be able to stand-alone. If additional details are necessary, they are added as a footnote to enable the reader to interpret the table.  Line 243: there’s a full-stop at the wrong place. Also, simplify the comparison between public and non-public facilities, it seems confusing.  The statement in line 255 needs to be in the discussion.  Line 257; check capitalizations. Also, if possible, avoid ‘respectively’ in your comparisons.  Line 263: avoid phrases like ‘crystal clear’; they have no relevance in scientific writing.  Lines 280-282; discussion. Same case applies to 290-294. | Revised  I prefer writing percent in a sentence. | Pg. 10-11 |
| I think table 4 should be either summarized or be a supplementary. | Revised | Pg. 13-14  line 296-297 |
| Qualitative: although the aim is to capture the verbatim responses from participants, the authors can assist in re-phrasing it for readers to understand. For instance, corona pandemic, COVID-19 can be added in parentheses. This should be done in a way that it doesn’t distort the original message | Re-phrasing may distort it is meaning | Pg. 16  line 362 |
| Discussion  Line 370: the authors have used compare in the same sentence twice.  Line 389: Be consistent with your spacing.  Lines 398 and 399: It is advisable to put reference at the end of the sentence, not in the middle. This makes reading easier.  Line 404: Were primary care facilities defined in the methods, and then linked with the KEPH structure/ If not, please review. | Revised | Pg. 17-18  line |
| Conclusion  The conclusion is too long and feels like a repetition of the discussion. The authors should use this section to summarize their key findings and through in some recommendations.  Study limitations: this should come before the acknowledgements. | Revised | Pg. 19-20  Line 433-450 |
