# Supplementary material for: Health systems’ capacity in availability of human resource for health towards implementation of Universal Health Coverage in Kenya
Source: PLoS One. 2024 Jan 30;19(1):e0297438. doi: 10.1371/journal.pone.0297438 (PMC10826929; doi:10.1371/journal.pone.0297438)
Supplement: Attachment — Submitted filename: Response to Reviewers.docx [file pone.0297438.s005.docx]

**Response to Reviewers**

| **Reviewer #3:** | |  |
| --- | --- | --- |
| **Comments** | **Response** | **Page; Line** |
| P 3; L 81-82: Reference for this statement? | Referenced | P3; L82 |
| P 4; L 92: Missing word - 'number'? | Highlighted | P4; L91 |
| P 6; L 151: Replace 'interviewed' with 'conducted' | Revised | P 6; L 150 |
| P 11; L 269: '.... in rural areas have meet staffing norms ........? | Revised | P11; L268-269 |
| P 14; L 301: Check heading - 'carders' | Corrected | P14; L300 |
| P 14; L 311: Check heading - 'RH' | Revised | P14; L310 |
| P 16; L 359-360: 'The overall HRH availability index of 17.2 percent' - against what? | Against 100% | P16; L358-359 |
| P 17; L 362: Insert period (.) after 'healthcare services' | Inserted | P17; L361 |
| P 17; L 365-366: 'WHO recommended minimum'. - Mention what is the WHO recommendation; and what is the percentage of deficiency vis-a-vis the recommendation. | Revised | P17; L365-366 |
| P 17; L 372: Comparable HRH available indexes - what are they? Menton or give reference to 'Table' if referred to in the Table | Referenced | P17; L373 |
| P 18; L 409: '.... had the necessary' - Look for missing word(s). | Revised | P18; L409 |
| P 22-23: References - (a) Author names remain incomplete or incorrect. (b) Inappropriate way of abbreviating organizational author (e.g., WHO). | Revised | P 22-23 |
